# Serum Soluble (Pro)Renin Receptor Levels in Maintenance Hemodialysis Patients

**DOI:** 10.1371/journal.pone.0158068

**Published:** 2016-07-01

**Authors:** Yoshifumi Amari, Satoshi Morimoto, Fumitaka Nakajima, Takashi Ando, Atsuhiro Ichihara

**Affiliations:** 1 Department of Medicine II, Endocrinology and Hypertension, Tokyo Women’s Medical University, Tokyo, Japan; 2 Department of Nephrology, Moriguchi Keijinkai Hospital, Osaka, Japan; The University of Manchester, UNITED KINGDOM

## Abstract

The (pro)renin receptor [(P)RR] is cleaved by furin to generate soluble (P)RR [s(P)RR], which reflects the status of the tissue renin-angiotensin system. Hemodialysis patients have advanced atherosclerosis. The aim of this study was to investigate the relationships between serum s(P)RR levels and background factors, including indices of atherosclerosis, in hemodialysis patients. Serum s(P)RR levels were measured in hemodialysis patients and clearance of s(P)RR through the membrane of the dialyzer was examined. Furthermore, relationships between serum s(P)RR levels and background factors were assessed. Serum s(P)RR levels were significantly higher in hemodialysis patients (30.4 ± 6.1 ng/ml, n = 258) than those in subjects with normal renal function (21.4 ± 6.2 ng/ml, n = 39, P < 0.0001). Clearance of s(P)RR and creatinine were 56.9 ± 33.5 and 147.6 ± 9.50 ml/min, respectively. Serum s(P)RR levels were significantly higher in those with ankle-brachial index (ABI) of < 0.9, an indicator of severe atherosclerosis, than those with ABI of ≥ 0.9 (32.2 ± 5.9 and 30.1 ± 6.2 ng/ml, respectively, P < 0.05). An association between low ABI and high serum s(P)RR levels was observed even after correction for age, history of smoking, HbA1c, and LDL-C. Serum s(P)RR levels were significantly higher in hemodialysis patients when compared with subjects with normal renal function, although s(P)RR is dialyzed to some extent, but to a lesser extent than creatinine. High serum s(P)RR levels may be associated with atherosclerosis independent of other risk factors, suggesting that serum s(P)RR could be used as a marker for atherosclerotic conditions in hemodialysis patients.

## Introduction

The tissue renin-angiotensin system (RAS), unlike the circulating RAS, which plays an essential role in regulating blood pressure (BP) and electrolyte balance [1], is associated with tissue-specific functions involved in cell growth, fibrosis, and inflammation in various organs [1, 2]. The activation of the tissue RAS is believed to cause progression of cardiovascular diseases. Prorenin, a nonactivated precursor of renin, is activated by binding to the (pro)renin receptor [(P)RR] and can carry out the conversion of angiotensinogen to angiotensin I like renin [3]. (P)RR, a receptor for renin and prorenin plays an important role in regulating the tissue RAS [3]. (P)RR, which consists of 350 amino acids with a single transmembrane domain, is cleaved by furin to generate soluble (P)RR [s(P)RR], which is secreted into the extracellular space and found in blood [4]. These findings suggest that s(P)RR can serve as a biomarker reflecting the tissue RAS status [5, 6].

Hemodialysis (HD) patients have poor prognosis due to increased prevalence of cardiovascular diseases [7, 8]. Although it is possible that activation of the tissue RAS by (P)RR is associated with this condition, the pathophysiology and clinical significance of s(P)RR in patients with maintenance HD therapy remain unclear. On the basis of these background findings, the aims of this study are roughly classified into the following three: 1) to determine serum s(P)RR levels in HD patients and to compare them with subjects with normal renal function, 2) to calculate the clearance and reduction rate of s(P)RR levels, and 3) to assess the relationships between serum s(P)RR and background factors, including indicators of atherosclerosis.

## Materials and Methods

### Study subjects

The study followed the Declaration of Helsinki and good clinical practice guidelines, and was approved by the ethical committee of Tokyo Women’s Medical University (approval #: 2703). All participants were enrolled after obtaining written informed consents". The subjects were outpatients on maintenance dialysis at Kadoma Keijinnkai Clinic, Neyagawa Keijinnkai Clinic, and Moriguchi Keijinnkai Clinic in Kadoma City, Japan, and those with normal renal function at Tokyo Women’s Medical University Hospital, Tokyo, Japan. A total of 258 maintenance HD patients and 39 subjects with normal renal function, whose estimated glomerular filtration rate (eGFR) calculated using the equation [9]:

$$eGFR\;\left(ml/min\;per\;1.73\;m^{2}\right)=194\times creatinine^{-1.094}\times age^{-0.287}\left(\times 0.739\;if\;female\right)$$

was higher than 90 ml/min/1.73 m^2^, were recruited from the aforementioned institutions between March and May 2013.

### Background factors

At enrollment, information was collected on age, gender, diabetes mellitus (DM) [10], duration of HD, smoking status, existence of past history of cardiovascular diseases, and body mass index (BMI). Five kinds of systolic BP (sBP) values were measured on the 1st dialysis day of the week. The first was sBP at the start of dialysis. The second was the highest sBP during dialysis. The third was the lowest sBP during dialysis. The fourth was the difference between the highest and lowest values. The fifth was sBP at the end of dialysis. Post-dialysis values of cardiothoracic ratio (CTR) were obtained on the 1st dialysis day of the week. The Kt/V was calculated on the 1st dialysis day of the week using the following equation, the formula of Daugirdas [11]:

$$Kt/V=-\;Ln\;\{\frac{post-dialysis\;value\;of\;BUN}{pre-dialysis\;value\;of\;BUN}–0.008\times dialysis\;time+(4-3.5\times \frac{post-dialysis\;value\;of\;BUN}{pre-dialysis\;value\;of\;BUN})\times amount\;of\;drainage\;/\;post-dialysis\;body\;weight\}$$

### Blood examinations

Non-fasting blood samples were taken while patients were lying in bed at supine position after at least 15 minutes of rest on the 1st dialysis day of the week. Pre-dialysis values of hemoglobin (Hb), hemoglobin A1c (HbA1c), creatinine (Cre), low-density lipoprotein cholesterol, (LDL-C), high-density lipoprotein cholesterol (HDL-C), triglyceride (TG), albumin (Alb), C-reactive protein (CRP), calcium (Ca), inorganic phosphorus (IP), intact parathyroid hormone (i-PTH), uric acid (UA), plasma renin activity (PRA), and plasma aldosterone concentration (PAC), and post-dialysis values of human atrial natriuretic peptide (hANP) and brain natriuretic peptide (BNP) were measured by conventional methods at an external testing laboratory (Kishimoto, Inc., Tomakomai City, Japan). Pre-dialysis serum levels of s(P)RR were measured using an enzyme-linked immunosorbent assay (ELISA) kit (Takara Bio Inc, Otsu City, Japan) consisting of a solid-phase sandwich ELISA with antibodies highly specific for each protein [12]. Post-dialysis serum s(P)RR and Cre levels were measured in one part of patients who underwent adequate dialysis therapy (Kt/V > 1.0) 3 times a week, in order to calculate the clearances and reduction rates of these values.

### Western blotting and immunoprecipitation analysis

HD waste water was obtained 15 min after start of HD in 3 patients. Ten times concentrates of hemodialysis waste water, Chinese hamster ovary cells transfected with cDNA for (P)RR [13] as a positive control, and dialysate (LYMPAC TA1, Nipro Corp, Osaka, Japan) as a negative control were lysed with a solution containing 2% SDS, 10% glycerol, 50 mM Tris-HCI (PH 6.8), and 100 mM dithiothreitol, and boiled and subjected to a Western blot analysis by using 93A1B, an anti-(P)RR antibody, supplied by the soluble (P)RR ELISA kit [13]. The reactivity of the antibody was assessed by immunoprecipitation Western blotting. The HD waste water and positive and negative control samples were incubated with 93A1B and then Protein G Sepharose (GE Healthcare Japan) was added. After further incubation, they were centrifuged and the resulting pellets were washed three times. The pellets were subsequently lysed and Western blot analysis was performed using 93A1B.

### Physiological function tests

#### Echocardiography

Echocardiography was performed on a non-dialysis day as previously described [14] using the Vivid S6 System (GE Healthcare, Milwaukee, WI, USA), and cardiac functions, including left ventricular ejection fraction (LVEF), a marker of contractile activity, left ventricular mass index (LVMI), a marker of cardiac hypertrophy, diameter of inferior vena cava (IVC) or left atrial internal dimension in systole (LADs), markers of blood fluid volume, and E over e-prime (E/e’), a marker of left ventricular diastolic function, were estimated.

#### Carotid intima-media thickness

Carotid ultrasound examinations of the common carotid artery, bulb, and internal carotid artery were performed bilaterally as described previously [15] using the Nemio 30 Ultrasound System (Toshiba Medical Systems Co., Ltd, Tochigi, Japan) on a non-dialysis day.

#### Ankle-brachial index and baPWV

The ankle-brachial index (ABI) and brachial-ankle pulse wave velocity (baPWV) values were measured on a non-dialysis day using the volume-plethysmographic apparatus PWV/ABI (Omron Healthcare Co., Ltd, Kyoto, Japan) in accordance with a methodology previously described [15]. The lower and higher values were accepted as the ABI and baPWV values, respectively. BaPWV cannot be estimated properly when ABI is less than 0.9 because arterial occlusion retards baPWV [16]. Therefore, patients with ABI < 0.9 were excluded from the analysis of baPWV.

### CT

Body fat distribution and abdominal circumference were determined using computed tomography (CT) imaging on a non-dialysis day with the use of the 64 Rows Multislice CT Scanner (Aquilion 64, Toshiba, Tokyo, Japan). The subcutaneous fat area (SFA) and intra-abdominal VFA were measured at the level of the umbilicus. Abdominal circumference was measured at the level of the umbilicus by using the software Ziostation 2 (Ziosoft, Tokyo, Japan).

### Study protocols

Pre-dialysis serum levels of s(P)RR on the 1st dialysis day of the week were measured, and these values were compared with those of subjects whose renal functions were normal. Relationships between s(P)RR levels and background factors were examined by single or multiple regression analyses. Immunoprecipitation Western blotting was performed to detect s(P)RR in hemodialysis waste water. The clearance and reduction rate of s(P)RR on the 1st dialysis day of the week were calculated using the following equations [17, 18]:

$$Clearance=\frac{\left(Qb-in\times Cb-in\right)-\left(Qb-out\times Cb-in\right)}{Cb-in}\left[ml/min\right]$$;

Qb-in: blood inflow into dialyzer; Cb-in: serum level of s(P)RR before passing dialyzer; Qb-out: blood outflow from dialyzer; Cb-out: serum level of s(P)RR after passing dialyzer.

$$Reduction\;rate=\frac{Cb-pre-Cb-post}{Cb-pre}\times 100\;[\%]$$;

Cb-pre: pre-dialysis serum level of s(P)RR; Cb-post: post-dialysis serum level of s(P)RR.

### Statistical analysis

All values are shown as the mean ± standard deviation (SD). Single regression analyses were performed to determine the correlation between serum s(P)RR levels and background factors. Multiple regression analyses were used to identify possible determinants. Non-paired Student’s t-test and χ^2^-test were applied to compare the two groups. The level of significance was defined as P < 0.05. All analyses were performed using Statview 5.0 statistical software (SAS Institute) an Jmp pro 12 statistical software (SAS institute).

## Results

### Characteristics of the study subjects

Table 1 details the background factors, comprising the preceding five kinds of sBP values, post-dialysis values of CTR, and Kt/V; blood data; data from the physiological function tests; and CT imaging data. With respect to antihypertensive treatment, 190 patients were treated with calcium channel blockers (CCBs) and 198 patients with RAS inhibitors, such as angiotensin receptor blockers (ARBs), angiotensin-converting enzyme inhibitors, and renin inhibitors.

**Table 1:** Characteristics of the study subjects.

**Background factors**		Calcium (mg/dl)	8.9±0.5
Age (y.o.)	67±12	Inorganic phosphorus (mg/dl)	5.2±1.2
Gender (male/female)	146/112	Intact parathyroid hormone (pg/ml)	143.4±85.0
Primary disease (Diabetes mellitus [DM] / non DM)	123/135	Uric acid (mg/dl)	7.2±1.3
Duration of hemodialysis therapy (months)	63±55	PRA (ng/ml)	3.9±5.6
Smoking history (yes/no)	115/143	PAC (pg/ml)	12.2±21.6
Past history of cardiovascular diseases (yes/no)		hANP (PG/ML)	69.3±107.9
myocardial infarction	59/199	BNP (pg/ml)	264.5±404.2
stroke	50/208	**Physical function tests**	
peripheral artery disease	8/250	Echocardiography	
Body mass index (kg/m^2^)	22.2±3.7	LVEF (%)	67.5±10.2
Systolic blood pressure during dialysis (mmHg)		LVMI	178.5±51.3
at start (average values of last three times)	149±20	IVC (mm)	13.4±4.0
highest values	160±21	LADs (mm)	41.0±5.2
lowest values	125±21	E/e'	19.5±7.5
difference between highest and lowest values	35±21	Carotid ultrasound examination	
at end	140±21	max carotid IMT (mm)	1.0±0.4
CTR (%)	52.3±5.3	ABI	
Kt/V	1.35±0.24	lower values	1.08±0.20
**Blood tests**		average values	1.13±0.19
Hemoglobin (g/dl)	10.8±1.0	≥ 0.9 / < 0.9	205 / 47
Hemoglobin A1c (%)	5.3±0.9	baPWV (cm/s)	
Creatinine (mg/dl)	9.59±2.54	higher values	1980±602
LDL cholesterol (mg/dl)	89±29	average values	1918±535
HDL cholesterol (mg/dl)	47±15	**Abdominal CT**	
Triglyceride (mg/dl)	103±66	Visceral fat area (cm^2^)	84.4±58.8
Albumin (g/dl)	3.7±0.3	Subcutaneous fat area (cm^2^)	120.2±74.8
CRP (mg/dl)	0.25±0.39	Abdominal circumference (cm)	83.6±10.4

CTR: cardiothoracic ratio, CRP: C-reactive protein, PRA: plasma renin activity, PAC: plasma aldosterone concentration, hANP: human atrial natriuretic peptide, BNP: brain natriuretic peptide, LVEF: left ventricular ejection fraction, LVMI: left ventricular mass index, IVC: inferior vena cava, LADs: left atrial internal dimension in systole, E/e': E over e-prime, IMT: intima-media thickness, ABI: ankle-brachial index, baPWV: brachial-ankle pulse wave velocity

### Serum s(P)RR levels

The average serum s(P)RR level in HD patients was 30.4 ± 6.1 ng/ml (n = 258). In 39 subjects (18 males) with normal renal function as a control group, there were 15 patients with diabetes mellitus, but no patients with cardiovascular diseases. The average age and eGFR were 64 ± 7 y.o. and 102.4 ± 13.4 ml/min/1.73 m^2^, respectively. The average serum s(P)RR level was 21.4 ± 6.2 ng/ml. Although age, gender, and prevalence of diabetes mellitus were not significantly different between patients undergoing HD and subjects with normal renal function, serum s(P)RR values were significantly higher in HD patients (P < 0.0001).

In HD patients, serum s(P)RR levels were slightly but significantly higher in females (31.3 ± 6.3 ng/ml, n = 112) than in males (29.8 ± 5.9, n = 146) (P < 0.05). There were other factors correlated with gender, such as smoking status, which was significantly higher in males (95 of 146 patients) than in females (20 of 112 patients) (P < 0.001). In addition, CTR (51.0 ± 4.9 vs 54.0 ± 5.3%, P < 0.001), Kt/V (1.44 ± 0.25 vs 1.28 ± 0.20, P < 0.001), LDL-C (95 ± 31 vs 84 ± 27 mg/dl, P < 0.01), and SFA (141.0 ± 79.7 vs 103.6 ± 66.4 cm^2^, P < 0.001) were significantly higher, and Cre (8.76 ± 2.42 vs 10.24 ± 2.45 mg/dl, P < 0.001) was significantly lower in females than in males. Multiple regression analysis, testing gender and SFA as independent variables, showed that SFA levels (β = 0.148, P < 0.05) but not gender were significantly correlated with serum s(P)RR levels (R^2^ = 0.035 and P < 0.05 for entire model). There were no significant differences in serum s(P)RR levels between patients with DM (30.1 ± 6.0 ng/ml, n = 123) and those without DM (30.7 ± 6.2, n = 135), between patients with (30.0 ± 6.5, n = 115) and without (30.8 ± 5.7, n = 143) smoking history, between patients with (30.3 ± 5.8, n = 59) and without (30.5 ± 6.2, n = 199) myocardial infarction, between patients with (30.2 ± 7.3, n = 50) and without (30.5 ± 5.8, n = 208) stoke, and between patients with (26.8 ± 4.7, n = 8) and without (30.5 ± 6.1, n = 250) angiographically confirmed peripheral artery disease (including 6 patients who had undergone lower limb amputations and 2 patients who had undergone percutaneous transluminal angioplasty but not amputation). s(P)RR levels were significantly lower in patients treated with RAS inhibitors (29.8 ± 5.7 ng/ml, n = 198) than in those without these drugs (32.6 ± 6.8, n = 60) (P < 0.01), and were significantly lower in patients treated with CCBs (29.9 ± 5.7 ng/ml, n = 190) than in those without these drugs (32.0 ± 6.9, n = 68) (P < 0.05). No significant differences in s(P)RR levels were noted between patients with or without other antihypertensive drugs, including β-blockers and diuretics.

### Detection of s(P)RR in hemodialysis waste water

**Fig 1:**
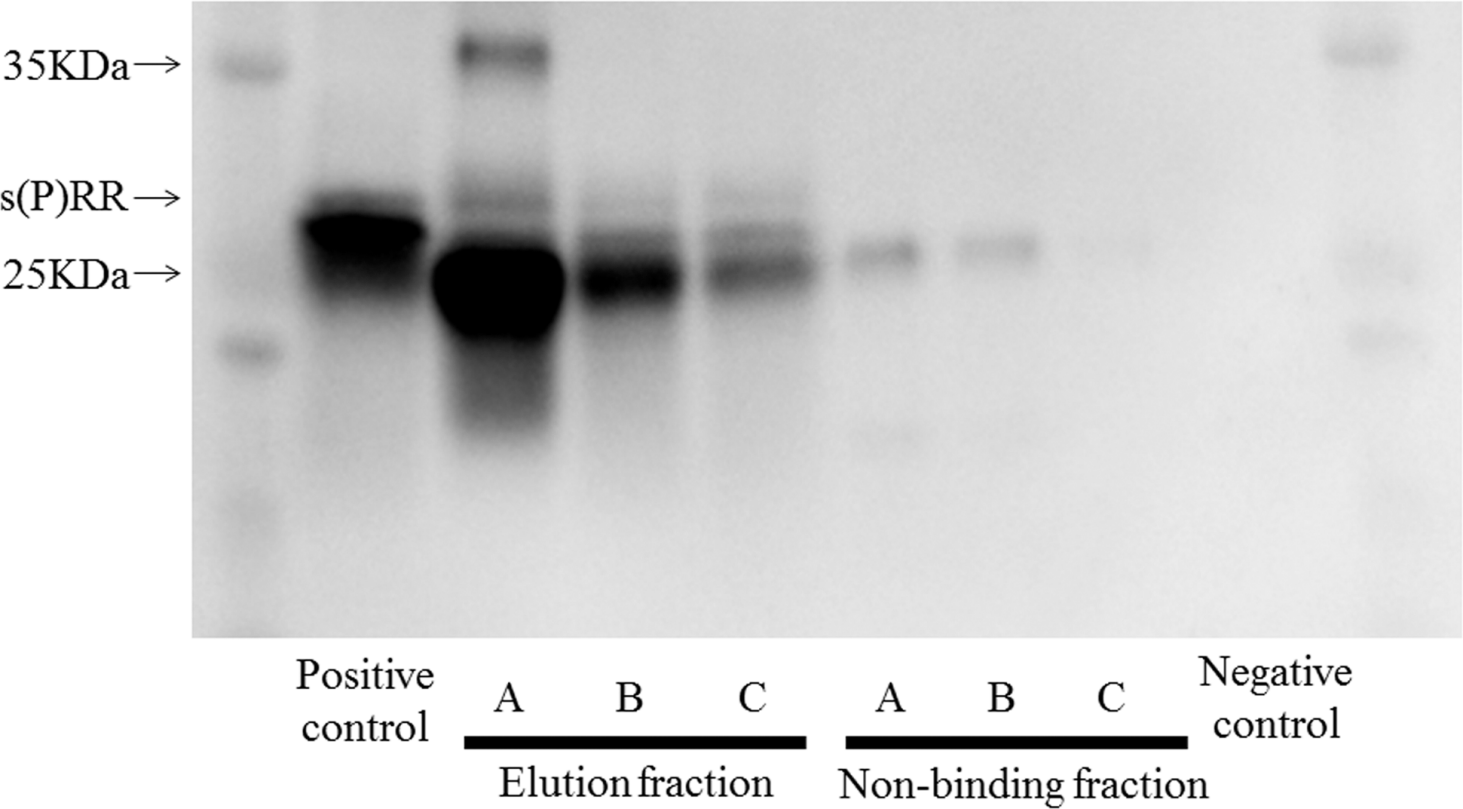
Western blotting and immunoprecipitation analysis. Identification of s(P)RR in hemodialysis waste water in 3 patients (A, B, and C). IgG, immunoglobulin G.

The results of immunoprecipitation Western blotting are shown in Fig 1. In all 3 hemodialysis waste water samples, a band indicating s(P)RR was detected.

### Clearance and reduction rate of s(P)RR through hemodialysis therapy

The clearance of s(P)RR and Cre through the membrane of a dialyzer were calculated to be 56.9 ± 33.5 and 147.6 ± 9.5 ml/min, respectively, in 20 patients (19 were on HD and 1 was on hemodiafiltration), with the mean blood flow of 211.0 ± 20.0 ml/min, the mean dialysis time of 3.6 ± 0.5 hours, and the mean Kt/V of 1.42 ± 025. The reduction rates of s(P)RR and Cre in one dialysis treatment were 10.1 ± 22.6 and 65.6 ± 4.5%, respectively, in 37 patients (26 were on HD and 11 were on hemodiafiltration), with the mean blood flow of 221.6 ± 20.2 ml/min, mean dialysis time of 3.6 ± 0.5 hours, and the mean Kt/V of 1.52 ± 0.18. These data indicated that s(P)RR is dialyzed to some extent, but to a lesser extent than Cre by adequate dialysis therapy.

### Correlation between background factors, blood data, physiological function tests, and CT imaging data and serum s(P)RR levels

CTR showed a significantly positive relationship with serum s(P)RR levels. Among the blood data, Hb levels showed a weak but significantly positive relationship with serum s(P)RR levels. TG, CRP, and IP levels showed significantly positive relationships with serum s(P)RR levels. No physiological function test data correlated with s(P)RR levels. Among the CT imaging data, VFA and SFA levels showed significantly positive relationships with serum s(P)RR levels (Table 2).

**Table 2:** Single regression analyses with serum soluble (pro)renin receptor levels.

	r	P		r	P
Background factors			Calcium	0.024	N.S.
Age	0.101	N.S.	Inorganic phosphorus	0.163	< 0.01
Duration of hemodialysis therapy	0.031	N.S.	Intact parathyroid hormone	0.117	N.S.
Smoking history	0.087	N.S.	Uric acid	0.081	N.S.
Past history of cardiovascular diseases			PRA	0.023	N.S.
myocardial infarction	-0.01	N.S.	PAC	0.022	N.S.
stroke	-0.019	N.S.	hANP	0.081	N.S.
peripheral artery disease	-0.106	N.S.	BNP	0.053	N.S.
Body mass index	0.072	N.S.	**Physical function tests**		
Systolic blood pressure during dialysis			Echocardiography		
at start	-0.191	< 0.01	LVEF	-0.051	N.S.
highest value	-0.186	< 0.01	LVMI	-0.074	N.S.
lowest value	-0.182	< 0.01	IVC	-0.100	N.S.
difference between highest and lowest values	-0.006	N.S.	LADs	-0.023	N.S.
at end	-0.189	< 0.01	E/e'	-0.042	N.S.
CTR	0.139	< 0.05	Carotid ultrasound examination		
Kt/V	0.079	N.S.	max carotid IMT	0.003	N.S.
**Blood tests**			ABI		
Hemoglobin	0.133	< 0.05	lower value	-0.069	N.S.
Hemoglobin A1c	-0.081	N.S.	average value	-0.072	N.S.
Creatinine	-0.081	N.S.	baPWV		
LDL cholesterol	0.048	N.S.	higher value	0.080	N.S.
HDL cholesterol	-0.072	N.S.	average value	0.077	N.S.
Triglyceride	0.266	< 0.01	**Abdominal CT**		
Albumin	-0.072	N.S.	Visceral fat area	0.184	< 0.01
CRP	0.170	< 0.01	Subcutaneous fat area	0.169	< 0.01
Log CRP	0.267	< 0.001	Abdominal circumference	0.111	N.S.

CTR: cardiothoracic ratio, CRP: C-reactive protein, PRA: plasma renin activity, PAC: plasma aldosterone concentration, hANP: human atrial natriuretic peptide, BNP: brain natriuretic peptide, LVEF: left ventricular ejection fraction, LVMI: left ventricular mass index, IVC: inferior vena cava, LADs: left atrial internal dimension in systole, E/e': E over e-prime, IMT: intima-media thickness, ABI: ankle-brachial index, baPWV: brachial-ankle pulse wave velocity

### Relationships between atherogenic factors and serum s(P)RR levels

Neither lower ABI nor average ABI were significantly associated with serum s(P)RR levels (Table 2). On the other hand, when the subjects were divided into those with ABI < 0.9, an indicator of severe atherosclerosis or obstruction of lower limb arteries, and those with ABI ≥ 0.9, serum s(P)RR levels were significantly higher in those with ABI < 0.9 (32.2 ± 5.9 ng/ml) than those with ABI ≥ 0.9 (30.1 ± 6.2) (P < 0.05), suggesting an interaction between severe atherosclerosis and serum s(P)RR levels. It is well known that age, smoking status, hypertension, impaired glucose metabolism, and dyslipidemia are risk factors of atherosclerosis. Multiple regression analyses testing age, smoking status, and s(P)RR, sBP, HbA1c, LDL-C and CRP levels as independent variables revealed that serum s(P)RR levels in addition to age and HbA1c levels were negatively correlated with ABI, suggesting the association between serum s(P)RR levels and ABI of < 0.9 is independent of these risk factors (Table 3).

**Table 3:** Multiple regression analyses with ABI.

	β	P
s(P)RR	-0.158	< 0.05
Age	-0.186	< 0.01
Smoking history	0.015	N.S.
Systolic blood pressure	-0.072	N.S.
Hemoglobin A1c	-0.169	< 0.01
LDL-cholesterol	0.010	N.S.
CRP	-0.025	N.S.
	R^2^ = 0.089, P < 0.01	
	for entire model	

CRP: C-reactive protein

### Relationships between BP-related factors and serum s(P)RR levels

All sBP values except difference between the highest and lowest values were significantly negatively correlated with s(P)RR levels (Table 2). Because BP may be affected by antihypertensive treatment or body fluid volume, multiple regression analyses were performed to determine if the relationship between BP and s(P)RR levels was independent of these factors (Table 4). In Model 1, where the independent variables were sBP, treatment with RAS inhibitors, treatment with CCBs, and hANP as independent variables, only sBP was related to s(P)RR. Also, in Models 2 and 3, where independent variables, IVC and CTR, were added as substitutes for hANP, respectively, only sBP was correlated with s(P)RR.

**Table 4:** Multiple regression analyses with s(P)RR.

	Model 1		Model 2		Model 3	
	β	P	β	P	β	P
Systolic blood pressure	-0.156	< 0.05	-0.144	< 0.05	-0.153	< 0.01
ARB therapy	-0.131	N.S.	-0.138	N.S.	-0.125	N.S.
CCB therapy	-0.051	N.S.	-0.058	N.S.	-0.043	N.S.
hANP	0.025	N.S.	—	—	—	—
IVC	—	—	-0.088	N.S.	—	—
CTR	—	—	—	—	0.093	N.S.
	R^2^ = 0.066, P < 0.01		R^2^ = 0.069, P < 0.01		R^2^ = 0.058, P < 0.01	
	for entire Model 1		for entire Model 2		for entire Model 3	

ARB: angiotensin receptor blocker, CCB: calcium channel blocker, hANP: human atrial natriuretic peptide, IVC: inferior vena cava, CTR: cardiothoracic ratio

### Relationships between obesity-related factors and s(P)RR levels

There were significant positive relationships between VFA or SFA and serum s(P)RR levels (Table 2). To determine which obesity-related factor(s) is(are) independently associated with s(P)RR, stepwise regression analysis testing VFA, SFA, waist circumference, and BMI and gender as independent variables was performed using forward method. VFA and gender were selected as suitable independent variables by calculating Akaike Information Criterion (AIC). At adjusted multiple regression analysis, only VFA was shown to be significantly positively correlated with s(P)RR (Table 5).

**Table 5:** Multiple regression analyses with s(P)RR.

	β	P	VIF
Visceral fat area	0.215	< 0.05	1.002
Gender	0.246	< 0.05	1.002
	R^2^ = 0.046, P < 0.01		
	for entire model		

VIF: Variance Inflation Factor

## Discussion

The present study demonstrated five major findings regarding serum s(P)RR levels in maintenance HD therapy. First, serum s(P)RR levels were significantly higher in HD patients when compared with age-matched subjects with normal renal function. Second, serum s(P)RR levels were positively correlated with CTR, Hb, TG, CRP, IP, VFA, and SFA, and were negatively correlated with sBP. Third, serum s(P)RR levels were significantly higher in patients with ABI < 0.9 than those with ABI ≥ 0.9, and this association was observed even after correction for atherogenic factors. Fourth, sBP values were negatively correlated with s(P)RR even after correction for RAS inhibition, calcium channel blockade, and body fluid volume. Finally, VFA was positively correlated with s(P)RR even after correction for other obesity-related factors.

### Serum s(P)RR levels

Serum s(P)RR levels were significantly higher in HD patients when compared with subjects with normal renal function. While the reason for this remains unclear, there are several possible explanations. First, elevated serum s(P)RR levels may be simply due to a decrease or loss of excretion from the kidney. Second, elevated serum s(P)RR levels may reflect an increase in expression of (P)RR in the kidneys, which could in turn increase the local production of angiotensin II by activating prorenin. Increased expression of (P)RR was observed in CKD rats [19]. We have reported that a (P)RR blocker handle region peptide inhibited the development and progression of renal damage in hypertensive rats [20]. Urinary protein excretion was reported to be significantly positively related with serum s(P)RR levels in CKD patients [21]. Therefore, increased expression of (P)RR in the kidney might lead to both elevated serum s(P)RR levels and renal dysfunction. Thirdly, increased serum s(P)RR levels might be the result of enhanced cleavage of (P)RR by furin. (P)RR plays a physiological role in the assembly and function of vacuolar H^+^-ATPase (V-ATPase), an ATP-dependent proton pump that transports protons across plasma membranes and acidifies intracellular compartments and has an important role in autophagy [22]. It is possible that increased cleavage of (P)RR inhibits (P)RR-dependent V-ATPase and impairs autophagy in the kidney to progress kidney damage. Further studies are needed to investigate these issues.

### Relationship between gender and serum s(P)RR levels

It has been reported that s(P)RR levels are not significantly correlated with gender [6, 23]. In contrast, in this study, serum s(P)RR levels were significantly higher in female than male patients. In addition, SFA was significantly higher in females (141.0 ± 79.7 cm^2^) than in males (103.6 ± 66.4) (P < 0.001). Multiple regression analysis, testing gender and SFA as independent variables, showed that SFA but not gender was significantly correlated with serum s(P)RR levels, suggesting that elevated serum s(P)RR levels in females are due to increased SFA in them.

### Clearance and reduction rate of s(P)RR through hemodialysis therapy

It is unknown if s(P)RR is filtered through the membrane of a dialyzer. Albumin with the molecular weight of 66 kDa is dialyzed to some extent [24]. Thus, it is easy to speculate that s(P)RR with the molecular weight of 28 KDa is also dialyzed. Indeed, in this study, s(P)RR was detected in HD waste water (Fig 1). The clearance of s(P)RR was 56.9 ± 33.5 ml/min and the reduction rate of s(P)RR was 10.1 ± 22.6%. This is the first report to show that s(P)RR is dialyzed to some extent.

### Relationships between atherogenic factors and serum s(P)RR levels

The tissue RAS is associated with tissue-specific functions in various organs [25], and plays an essential role in fibrosis and inflammation in cardiovascular and renal tissues [1, 2]. ABI has recently been widely employed for screening arterial occlusive disease [26]. Fu et al. [27] reported relationships of ABI with left ventricular hypertrophy, coronary arterial lesions, and the mortality of HD patients. In this study, serum s(P)RR levels were significantly higher in patients with ABI of < 0.9 than those with ABI of ≥ 0.9. The association was observed even after correction for atherogenic factors (Table 3). Therefore, it is considered that serum s(P)RR levels are associated with severe atherosclerosis of the lower limbs independent of other risk factors. Additionally, it can be speculated that high serum s(P)RR levels could be a marker for progression of atherosclerosis. We are currently examining whether high serum s(P)RR levels can be a predictor of cardiovascular morbidity and mortality in HD patients.

### Relationships between cardiac functions and serum s(P)RR levels

Hirose T et al. [28] reported that gene expression of (P)RR was elevated in the heart and kidneys of chronic heart failure rats. We have reported that the long-term administration of a (P)RR blocker attenuated the development of cardiac fibrosis and hypertrophy [29]. These findings raise the possibility that (P)RR contributes to cardiac fibrosis and hypertrophy. However, in the present study, cardiac functions estimated by echocardiography were not significantly associated with serum s(P)RR levels. The reason for this result is unclear; however, other confounding factors that affect cardiac functions in HD patients may have unmasked the effects of s(P)RR. This issue should be addressed by further investigations.

### Relationships between blood pressure-related factors and serum s(P)RR levels

Hamada K et al. [21] reported that a significantly lower level of serum s(P)RR was observed in CKD patients treated with ARBs. Siragy et al. [30] reported that ARB valsartan significantly inhibited the renal expressions of (P)RR mRNA and protein in diabetic rats. In our study, serum s(P)RR levels were significantly lower in patients treated with RAS inhibitors or CCBs than in those without these drugs. Burckle´ et al. [31] reported that elevated BP and heart rate were observed in rats with human (P)RR gene overexpression, suggesting that (P)RR may have a certain role in BP regulation.

It has been reported that CKD patients with hypertension had significantly lower levels of s(P)RR than those without hypertension [21]. In the present study, sBP negatively correlated with serum s(P)RR levels even after correction for antihypertensive treatment and body fluid volume, which could affect BP. It is considered that s(P)RR levels are negatively related to BP due to unknown mechanisms other than these factors. The reason why parameters of blood pressures are negatively associated with serum s(P)RR needs to be discussed, because hypertension is considered as a major risk factor for cardiovascular events. Renin downregulates (P)RR expression by a process involving the transcription factor promyelocytic zinc finger protein [32]. Upregulation of renal renin and downregulation of renal (P)RR are observed after angiotensin-converting enzyme inhibition and change to a low-salt diet [33]. These facts raise the possibility that factors to compensate BP reduction such as increased renin expression might inhibit (P)RR expression to decrease serum s(P)RR levels. However, this remains just speculative and further studies are required to address this presumption.

### Relationships between obesity-related factors and s(P)RR levels

Serum s(P)RR levels were positively correlated with TG, suggesting the presence of interactions between dyslipidemia and serum s(P)RR levels (Table 2). Although non-fasting blood was obtained in this study, leading the possibility that this result might not be reliable, this association is supported by our previous data showing the relationship between serum levels of s(P)RR and TG in patients with hypertension as well [6]. VFA showed significantly positive relationships with serum s(P)RR levels independent of other factors related with obesity (Tables 2 and 5). Adipose tissue harbors all components of RAS, including (P)RR [34, 35]. Achard et al. [36] reported that overfeeding and a high-fat diet increased visceral fat mass and adipose (P)RR expression in rats. Therefore, it might be possible that patients with dyslipidemia have increased expression of (P)RR in adipose tissues that causes an elevation in serum (P)RR levels. However, this point should be investigated in detail by further studies.

## Limitations

Several limitations to the present study warrant mention. Firstly, the present data of HD patients may be modified by HD therapy, because s(P)RR is dialyzed to some extent. Secondly, this study is a cross-sectional study. The mechanisms by which serum s(P)RR levels are associated with background factors remain unclear. Whether s(P)RR is a biomarker for the status of atherosclerosis or whether it is the result of atherosclerosis remains unclear. Further study will be required to clarify the role of serum s(P)RR levels in HD patients and to determine the relationship between the prognosis of HD patients and serum s(P)RR levels.

## Conclusions

Serum s(P)RR levels were significantly higher in HD patients when compared with subjects with normal renal function. Serum s(P)RR levels should be interpreted carefully if the blood was obtained before or after HD because s(P)RR is dialyzed to some extent. High serum s(P)RR levels in HD patients were associated with severe atherosclerosis of the lower limbs independent of other risk factors. It could be possible that serum s(P)RR can be used as a marker for atherosclerotic conditions and a predictive marker for cardiovascular events in HD patients. Longitudinal follow-up studies to investigate the relationships between serum s(P)RR levels and mortality and morbidity of cardiovascular diseases are now in progress.
